# Waist-Height Ratio and the Risk of Severe Diabetic Eye Disease in Type 1 Diabetes: A 15-Year Cohort Study

**DOI:** 10.1210/clinem/dgab671

**Published:** 2021-09-11

**Authors:** Erika B Parente, Valma Harjutsalo, Carol Forsblom, Per-Henrik Groop

**Affiliations:** 1 Folkhälsan Institute of Genetics, Folkhälsan Research Center, Helsinki, Finland; 2 Research Program for Clinical and Molecular Metabolism, Faculty of Medicine, University of Helsinki, Helsinki, Finland; 3 Department of Nephrology, University of Helsinki and Helsinki University Hospital, Helsinki, Finland; 4 National Institute for Health and Welfare, Helsinki, Finland; 5 Department of Diabetes, Central Clinical School, Monash University, Melbourne, Victoria, Australia

**Keywords:** retinopathy, type 1 diabetes, body composition, waist-height ratio, nephropathy

## Abstract

**Context:**

Obesity prevalence has increased in type 1 diabetes (T1D). However, the relationship between body composition and severe diabetic eye disease (SDED) is unknown.

**Objective:**

To investigate the associations between body composition and SDED in adults with T1D.

**Methods:**

From 5401 adults with T1D in the Finnish Diabetic Nephropathy Study, we assessed 3468, and 437 underwent dual-energy X-ray absorptiometry for body composition analysis. The composite outcome was SDED, defined as proliferative retinopathy, laser treatment, antivascular endothelial growth factor treatment, diabetic maculopathy, vitreous hemorrhage, and vitrectomy. Logistic regression analysis evaluated the associations between body composition and SDED. Multivariable Cox regression analysis assessed the associations between the anthropometric measures and SDED. Subgroup analysis was performed by stages of albuminuria. The relevance ranking of each variable was based on the *z* statistic.

**Results:**

During a median follow-up of 14.5 (interquartile range 7.8-17.5) years, 886 SDED events occurred. Visceral/android fat ratio was associated with SDED [odds ratio (OR) 1.40, *z* = 3.13], as well as the percentages of visceral (OR 1.80, *z* = 2.45) and android fat (OR 1.28, *z* = 2.08) but not the total body fat percentage. Waist-height ratio (WHtR) showed the strongest association with the SDED risk [hazard ratio (HR) = 1.28, *z* = 3.73], followed by the waist (HR 1.01, *z* = 3.03), body mass index (HR 1.03, *z* = 2.33), and waist-hip ratio (HR 1.15, *z* = 2.22). The results were similar in normo- and microalbuminuria but not significant in macroalbuminuria. A WHtR ≥ 0.5 increased the SDED risk by 28% at the normo- and microalbuminuria stages.

**Conclusions:**

WHtR, a hallmark of central obesity, is associated with SDED in individuals with T1D.

Diabetic retinopathy (DR) is a common microvascular complication of diabetes, which may progress to severe stages and even to blindness. It is the fifth most common cause of blindness and visual impairment worldwide (1). From 1990 to 2015, the crude global prevalence of each cause of blindness and visual impairment decreased, except for DR, which increased (1). Also, given that the incidence of type 1 diabetes (T1D) increases by 4.2% annually among young adults (2), it is possible that in the future there will be an even higher number of individuals who have to cope with T1D.

The knowledge of modifiable risk factors, early diagnosis, and treatment are crucial to avoid the progression and the burden of DR. Although there are well-known risk factors for severe diabetic eye disease (SDED) in individuals with T1D (3-5), it is still unknown whether central obesity is related to SDED in those individuals. A few cross-sectional studies have been conducted in individuals with T1D to assess the relationship between body mass index (BMI) and DR, but the results are controversial (6,7). Furthermore, BMI may not reflect central obesity (8,9), which has been considered a risk factor for DR in individuals with type 2 diabetes (10,11).

Considering that the prevalence of obesity has increased along with T1D in the last decades (12), it is important to understand whether fat distribution, especially visceral fat, is a risk factor for SDED in this population. Thus, this study aimed to explore the associations between body composition and SDED in adults with T1D.

## Materials and Methods

### Study Design

This study included 2 different analyses. First, an observational prospective study was conducted to investigate the impact of anthropometric measures related to central obesity [waist-height ratio (WHtR), waist-hip ratio (WHR), and waist circumference (WC)] and BMI as a measure of general obesity on the risk of SDED in a large cohort of adults with T1D.

Second, a cross-sectional analysis was performed to investigate the association between body composition and the prevalence of any retinopathy except SDED or SDED. Furthermore, a similar cross-sectional analysis was performed to evaluate the association between WHtR (representing central obesity) and the Early Treatment of Diabetic Retinopathy Study (ETDRS) grading (13).

### Study Population

The Finnish Diabetic Nephropathy (FinnDiane) Study is a nationwide, prospective, multicenter (93 centers across Finland) study since 1997 that aims to identify risk factors for T1D complications, and recruitment of new participants is still ongoing.

For the longitudinal analysis, from a total of 5401 individuals with T1D in the FinnDiane cohort, 1933 individuals were excluded due to SDED at baseline. Thus, we assessed 3468 individuals for the occurrence of SDED. Then, since no anthropometric was associated with SDED in the macroalbuminuria stage, we limited the analyses to 3146 individuals with normo- and microalbuminuria from which 437 had their body composition evaluated by dual-energy X-ray absorptiometry (DXA), which was included in the regular FinnDiane study visit since 2011. Among all FinnDiane participants, we have 1319 individuals with ETDRS grading at baseline visit. Then, after excluding 551 individuals with SDED at baseline, we included 768 individuals in a sensitivity longitudinal analysis for the association between WHtR and SDED. The baseline visit occurred between 1997 and 2017 during which the participants underwent a thorough clinical examination, blood and urine samples were collected, and several questionnaires were completed by the participants. The same procedures were repeated at each follow-up visit. Type 1 diabetes was defined as age at onset of diabetes under 40 years and permanent insulin treatment initiated within a year from the diabetes diagnosis. The study protocol followed the principles of the Declaration of Helsinki as revised in 2000 and was approved by the Ethical Committee of Helsinki and Uusimaa Hospital District. Written informed consent was obtained from each FinnDiane participant, but it was not appropriate or possible to involve patients or the public in the design, conduct, reporting, or dissemination plans of our research.

### Diabetic Nephropathy Stages

Diabetic nephropathy (DN) stage was based on the individuals’ urinary albumin excretion rate (UAER) in timed overnight or 24-h urine (mg/24 h) collections. Normoalbuminuria was defined as a UAER <20 µg/min or <30 mg/24 h in at least 2 out of 3 consecutive urine samples. Microalbuminuria was defined as UAER ≥ 20 and <200 µg/min or ≥30 and <300 mg/24 h, and macroalbuminuria was defined as UAER ≥ 200 µg/min or ≥300 mg/24 h.

### Diabetic Retinopathy

The composite outcome was SDED, defined as proliferative DR (PDR), the initiation of laser treatment or antivascular endothelial growth factor (anti-VEGF), diabetic maculopathy, vitreous hemorrhage, and vitrectomy identified from the Care Register for Health Care until the end of 2017, whatever comes first. The DR classification at baseline was based on the FinnDiane questionnaire in which the participant as well as the attending physician answered the question of whether the participant had or did not have previous DR and/or had undergone laser treatment for diabetic eye disease. Furthermore, this information was later double-checked by a physician from the FinnDiane Study Group by reviewing the patient files for all potential information on retinal screening and ophthalmology consultations. Thus, participants were categorized into no retinopathy, any retinopathy except SDED, and SDED. In a subset of participants, ETDRS grading data were available for further sensitivity analysis, and they were classified at baseline according to the ETDRS grading as no retinopathy (ETDRS 10), mild non-PDR (NPDR; ETDRS 20 and 35), moderate NPDR (ETDRS 43 and 47), severe NPDR (ETDRS 53), and PDR (ETDRS 61-85) (13).

### Body Composition and Anthropometric Measurements

Body composition was assessed by DXA (GE Healthcare Lunar version 16, Madison, WI, USA) according to the manufacturer’s instructions, and visceral fat was measured by the CoreScan software (14) from 2015 to 2019 as part of the routine FinnDiane visits. The fat and lean mass were adjusted for the total body weight and are presented as percentages such as body fat mass percentage, android fat mass percentage, visceral fat mass percentage, body lean mass percentage, and appendicular lean mass percentage. The term *appendicular lean mass* refers to the lean mass of both legs and arms. BMI was calculated as total body weight (kilograms) divided by the square of the height (meters). WC was measured in centimeters by a stretch‐resistant tape at the horizontal plane midway between the superior iliac crest and the lower margin of the lowest rib. The hip circumference was measured with the same tape around the widest part over the great trochanters, and WHR and was calculated by dividing the WC by the hip circumference. The WHtR was calculated by dividing the WC by the height. Values <0.5 were considered normal for both sexes (15).

### Statistical Analyses

Data on categorical variables are presented as frequencies; continuous variables, as means (±SD) for normally distributed values; and otherwise, as medians [interquartile range (IQR)]. Between-group comparisons were performed with the χ ^2^ test for categorical variables, with analysis of variance for normally distributed continuous variables, and with the Mann-Whitney or Kruskal-Wallis test for nonnormally distributed continuous variables.

After excluding the individuals with SDED at baseline, a multivariable Cox regression analysis was used to assess the association between the anthropometric measures and the risk of SDED adjusted for baseline covariates such as age at onset of diabetes, duration of diabetes, sex, glycated hemoglobin A1c (HbA1c), systolic blood pressure, triglycerides, smoking, lipid-lowering medication, any retinopathy except SDED, estimated glomerular filtration rate (eGFR), and DN stages. Then, in a subset of 768 participants, a sensitivity analysis for the association between WHtR and the risk of SDED was performed using a similar model but replacing the covariate any retinopathy except SDED at baseline with ETDRS grading at baseline. Follow-up time was counted from the baseline visit until 1 of the components of SDED occurred, death, or the end of 2017. First analyses were done in the pooled population. However, the interaction terms between the DN stage, all anthropometric measures, and SDED were significant, indicating that the effect on the risk of SDED was dependent on the DN stage. Therefore, further analyses were performed separately according to DN stages. Since in the subgroup analyses by DN stages no anthropometric measure was associated with the risk of SDED at the macroalbuminuria stage, we used a final model that comprised of the pooled group of individuals with normo- and microalbuminuria. The relevance ranking of each variable was based on *z* statistics (3). Given that WHtR was the anthropometric measure most strongly associated with the risk of SDED, we performed a score ranking of WHtR and the other risk factors (HbA1c, age at onset of diabetes, duration of diabetes, triglycerides, systolic blood pressure, smoking, sex, lipid-lowering medication, any retinopathy except SDED, eGFR, and DN stages) using *z* statistics.

Since the interactions between sex and the anthropometric measurements or body composition variables were not significant, the analyses were conducted by pooling men and women together.

Finally, we used the %FINDCUT SAS macro tool to identify an optimal cutoff point for the WHtR to classify individuals at high risk *vs* low risk of SDED (16). After the establishment of the cutoff value, 2 groups were created, and the risk of SDED was compared between the groups.

In the cross-sectional analysis, a multinominal logistic regression model was used to evaluate the associations between body composition and any retinopathy except SDED or SDED, taking the no retinopathy group as the reference group. The model was adjusted for HbA1c, systolic blood pressure, triglycerides, smoking, lipid-lowering medication, eGFR, and DN stages. The same model was used in the cross-sectional analysis to evaluate the association between WHtR and baseline ETDRS grading.

All analyses were performed with the Statistical Analysis System version 9.4 (SAS Institute, Cary, NC, USA).

## Results

### Association Between Anthropometric Measures and SDED

In the longitudinal dataset including all 3468 individuals with T1D, the median age was 34.8 (IQR 25.7-45.1) years, 51.2% were female, and the median duration of diabetes was 15.3 (IQR 8.2-23.4) years. During a median follow-up of 14.5 (IQR 7.8-17.5) years, 886 incident cases of SDED occurred, giving an incidence rate of 25.6%. The baseline characteristics of all individuals according to the incidence of SDED are depicted in Table 1.

**Table 1.** Baseline clinical characteristics according to the incidence of severe diabetic eye disease

	All			Normo- and microalbuminuria		
	SDED (−)	SDED (+)	P-value	SDED (−)	SDED (+)	P-value
n (%)	2582 (74.4)	886 (25.6)		2380 (75.7)	766 (24.3)	
Women, %	52.8	46.5	<0.01	53.5	46.7	<0.001
Age, years	35.4 (26.6-45.8)	32.4 (23.9-43.5)	<0.0001	35.1 (26.4-44.9)	31.4 (23.4-43.2)	<0.0001
Age at onset of diabetes, years	18.0 (11.5-27.4)	14.2 (8.8-24.8)	<0.0001	18.1 (11.6-27.2)	14.1 (8.8-25.1)	<0.0001
Duration of diabetes, years	14.8 (7.4-23.7)	15.9 (10.4-22.3)	<0.0001	14.4 (7.3-23.3)	15.2 (10.0-21.3)	0.06
Height, cm	171.5 ± 9.4	171.4 ± 9.3	0.68	171.5 ± 9.4	171.5 ± 9.2	0.68
Weight, kg	72.3 (63.9-81.3)	74.5 (64.6-83.0)	<0.01	73.2 ± 13.0	74.4 ± 13.0	0.02
BMI, kg/m^2^	24.5 (22.5-26.6)	25.0 (22.7-27.6)	<0.0001	24.4 (22.5-26.5)	25.0 (22.5-27.5)	<0.001
WHR	0.85 ± 0.08	0.86 ± 0.08	<0.0001	0.85 ± 0.08	0.86 ± 0.08	<0.0001
WHtR	0.48 (0.45-0.52)	0.49 (0.46-0.54)	<0.0001	0.48 (0.45-0.52)	0.49 (0.46-0.53)	<0.0001
WC, cm	83.0 (76.0-90.0)	85.0 (78.0-93.5)	<0.0001	83.0 (76.0-90.0)	85.0 (78.0-93.0)	<0.0001
HbA1c, %	7.9 ± 1.29	9.2 ± 1.61	<0.0001	7.93 ± 1.29	9.16 ± 1.60	<0.0001
Systolic blood pressure, mmHg	130 ± 16	132 ± 16	<0.0001	128 (118-138)	130 (120-140)	<0.01
Diastolic blood pressure, mmHg	78±9	80 ± 9	<0.0001	78 (71-84)	80 (74-86)	<0.0001
Total cholesterol, mmol/L	4.74 ± 0.91	5.00 ± 0.95	<0.0001	4.65 (4.10-5.24)	4.90 (4.31-5.55)	<0.0001
HDL-cholesterol, mmol/L	1.41 ± 0.40	1.32 ± 0.38	<0.0001	1.37 (1.14-1.64)	1.27 (1.07-1.55)	<0.0001
Triglycerides, mmol/L	0.93 (0.70-1.28)	1.12 (0.85-1.55)	<0.0001	0.91 (0.70-1.27)	1.11 (0.83-1.51)	<0.0001
eGFR, mL/min/1.73 m^2^	101 (86-114)	100 (84-115)	0.28	100 ± 19	101 ± 20	0.21
Diabetic nephropathy stages, %			<0.0001			<0.0001
Normoalbuminuria	85.8	70.0		93.0	80.9	
Microalbuminuria	6.4	16.5		7.0	19.1	
Macroalbuminuria	2.8	8.5		-	-	
Nonclassified	5.0	5.1		-	-	
Any retinopathy except SDED, %	26.9	42.9	<0.0001	26.1	40.6	<0.0001
Lipid-lowering medication, %	8.4	7.9	0.16	7.7	6.3	0.20
Smoking history yes, %	42.6	50.4	<0.001	41.8	48.9	<0.001

Data on categorical variables are presented as frequencies; continuous variables are presented as means ± SD for normally distributed values and otherwise as medians (interquartile range). Between-group comparisons were performed with χ ^2^ test, *t*-test, and Mann-Whitney test, respectively.

Abbreviations: BMI, body mass index; eGFR, estimated glomerular filtration rate; HbA1c, glycated hemoglobin; HDL, high-density lipoprotein; SDED, severe diabetic eye disease; WC, waist circumference; WHR, waist-hip ratio; WHtR, waist-height ratio.

In the analysis including all individuals, the WHtR was the anthropometric measure most strongly associated with the risk of SDED [hazard ratio (HR) = 1.28 for 0.1 increase, *z* = 3.73], followed by WC (HR 1.01 for 1-cm increase, *z* = 3.03), BMI (HR 1.03 per 1-kg/m^2^ increase, *z* = 2.33), and WHR (HR 1.15 for 0.1 increase, *z* = 2.22) (Table 2). In the subgroup analysis by each eye disease outcome, WHtR was associated with maculopathy (HR 1.48, 95% CI 1.25-1.75, *P* < 0.0001) and PDR (HR 1.22, 95% CI 1.02-1.45, *P *= 0.03) with or without laser or anti-VEGF treatment but not with vitreous hemorrhage (HR 1.14, 95% CI 0.83-1.55, *P *= 0.42) with or without vitrectomy.

**Table 2.** Association between anthropometric measures and the risk of severe diabetic eye disease according to stages of diabetic nephropathy

	HR (95%CI)	z-value	P-value
All individuals, n = 3468			
WHtR, per 0.1 increase	1.28 (1.13-1.47)	3.7337	0.0002
WC, per 1-cm increase	1.01 (1.00-1.02)	3.0310	0.002
BMI, per 1-kg/m^2^ increase	1.03 (1.00-1.05)	2.3267	0.020
WHR, per 0.1 increase	1.15 (1.02-1.29)	2.2210	0.026
Normo- and microalbuminuria, n = 3146			
WHtR, per 0.1 increase	1.32 (1.15-1.52)	3.8562	0.0001
WC, per 1-cm increase	1.01 (1.00-1.02)	3.0360	0.002
BMI, per 1-kg/m^2^ increase	1.03 (1.01-1.06)	2.7325	0.006
WHR, per 0.1 increase	1.13 (1.00-1.29)	1.8892	0.059

Multivariable Cox regression model was adjusted for age at onset of diabetes, duration of diabetes, sex, glycated hemoglobin A1c, systolic blood pressure, triglycerides, smoking, lipid-lowering medication, any retinopathy except SDED, and estimated glomerular filtration rate. Diabetic nephropathy stage was also included as a covariate in the Cox regression model with all individuals.

Abbreviations: BMI, body mass index; WC, waist circumference; WHR, waist-hip ratio; WHtR, waist-height ratio.

In a sensitivity analysis including individuals with ETDRS grading at baseline, using the no retinopathy (ETDRS 10) as the reference group in a multinominal logistic regression model, WHtR was associated with increased odds of having NPDR (OR 1.91, 95% CI 1.06-3.47, *P *= 0.03) or PDR (OR 3.24, 95% CI 1.66-6.31, *P *= 0.0006) at baseline. Furthermore, the WHtR was also associated with the risk of SDED after adjusting for all covariates plus baseline ETDRS grading (HR 1.36, 95% CI 1.02-1.83, *P *= 0.039) in a subset of 768 individuals.

In the score ranking of the relevance of SDED risk factors, WHtR appeared in the sixth position (HR 1.25, 95% CI 1.09-1.42, *z* = 3.25). The top-ranking variable was HbA1c (HR 1.50, 95% CI 1.44-1.56, *z* = 18.09), followed by any retinopathy except SDED at baseline (HR 1.90, 95% CI 1.60-2.25, *z* = 7.41), the presence of albuminuria (HR 1.61, 95% CI 1.35-1.92, *z* = 5.34), age at onset of diabetes (HR 0.99, 95% CI 0.98-0.99, *z* = −3.60), and triglycerides (HR 1.19, 95% CI 1.11-1.28, *z* = 4.77].

### Subgroups by DN Stages

In the subgroup analysis by DN stages, WHR was no more associated with SDED in the individuals with normo- and microalbuminuria (Table 2). At the macroalbuminuria stage, no anthropometric measure was associated with SDED.

After excluding the individuals with macroalbuminuria and unknown stage of albuminuria at baseline, 3146 individuals remained for further analysis, of which 24.3% developed SDED during a median follow-up of 15.0 (IQR 8.4-17.6) years (Table 1). At baseline, the median age was 34.3 (25.3-44.4) years, 51.9% were women, and the median duration of diabetes was 14.7 (7.9-22.6) years. Among the individuals with normo- and microalbuminuria, at baseline 99.6% of those with obesity (BMI > 30 kg/m^2^), 69.1% of those with overweight (BMI ≥ 25 kg/m^2^ and <30kg/m^2^), and 10.7% of those with normal weight (BMI < 25 kg/m^2^) presented a WHtR ≥ 0.5. Baseline clinical characteristics according to the incidence of SDED in individuals with normo- and microalbuminuria are shown in Table 1.

**Figure 1.**
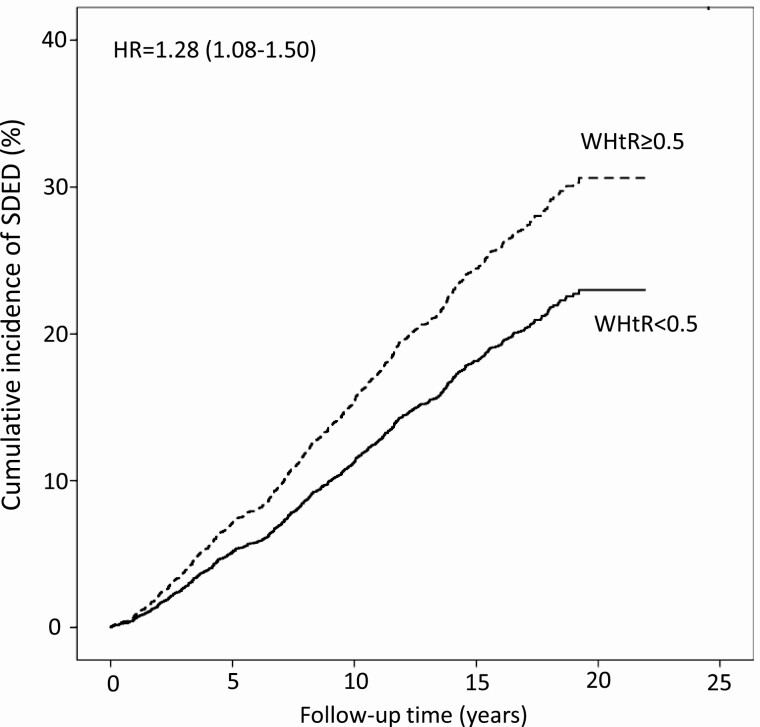
Cumulative incidence of severe diabetic eye disease in individuals with normo- and microalbuminuria according to the waist-height ratio threshold of 0.5. Abbreviations: HR, hazard ratio; WHtR, waist-height ratio.

Among those with normo- and microalbuminuria, the WHtR was the anthropometric measure most strongly associated with the risk of SDED (HR 1.32 per 0.1 increase, *z* = 3.86), followed by WC (HR 1.01 per 1-cm increase, *z* = 3.04) and BMI (HR 1.03 per 1-kg/m^2^ increase, *z* = 2.73) (Table 2). The results were similar when individuals with normo- or microalbuminuria were analyzed separately. The risk of SDED in the group with normo- and microalbuminuria was 28% higher (HR 1.28, 95% CI 1.08-1.50) in individuals with a WHtR ≥ 0.5 compared to the individuals with a WHtR < 0.5 (Fig. 1).

In the score ranking of the relevance of SDED risk factors, at the normo- and microalbuminuria stages WHtR appeared at the fifth position (HR 1.32, 95% CI 1.15-1.52, *z* = 3.86). HbA1c was again the most important risk factor (HR 1.53, 95% CI 1.46-1.61, *z* = 18.04), followed by any retinopathy except SDED at baseline (HR 2.02, 95% CI 1.68-2.42, *z* = 7.60), triglycerides (HR 1.20, 95% CI 1.11-1.29, *z* = 4.77), and the age at onset of diabetes (HR 0.98, 95% CI 0.97-0.99, *z* = −4.35).

### Association Between Body Composition and SDED

Individuals with SDED presented similar body weight and lean mass (total and appendicular lean mass), notwithstanding that they had a higher percentage of body fat mass, visceral fat mass, android fat mass, and a lower percentage of body lean mass and appendicular lean mass compared to those without SDED. Consequently, they had higher ratios of visceral and android fat to appendicular lean mass (Table 3).

**Table 3.** Body composition according to the prevalence of severe diabetic eye disease

	SDED (−)	SDED (+)	P-value
n (%)	293 (67)	144 (33)	
Total body weight, kg	74.4 (64.5-86.8)	77.5 (67.1-85.8)	0.34
Total body fat mass, kg	23.3 (17.4-29.1)	26.1 (19.2-33.4)	0.02
Total body fat mass, %	31.1 (25.8-36.4)	34.1 (27.6-40.8)	<0.01
Android fat mass, kg	1.79 (1.13-2.72)	2.24 (1.48-3.29)	<0.01
Android fat mass, %	2.40 (1.70-3.30)	3.00 (2.00-3.90)	<0.001
Visceral fat mass, kg	0.45 (0.18-1.02)	0.69 (0.40-1.50)	<0.0001
Visceral fat mass, %	0.62 (0.26-1.23)	0.93 (0.55-1.73)	<0.0001
Total body lean mass, kg	47.0 (41.4-56.5)	45.7 (42.0-54.3)	0.52
Total body lean mass, %	65.2 (60.3-70.3)	62.8 (55.8-68.5)	0.01
Appendicular lean mass, kg	20.8 (17.9-25.8)	20.0 (18.0-24.3)	0.24
Appendicular lean mass, %	28.9 (26.1-31.6)	26.8 (24.4-30.4)	<0.001
Visceral fat/android fat mass ratio	0.29 (0.14-0.42)	0.36 (0.24-0.49)	<0.0001
Visceral fat/appendicular lean mass ratio	0.02 (0.01-0.05)	0.04 (0.02-0.07)	<0.0001
Android fat/appendicular lean mass ratio	0.08 (0.06-0.12)	0.11 (0.07-0.15)	<0.0001

Data are presented as medians (interquartile range). Between-group comparisons were performed with Mann-Whitney test.

Abbreviation: SDED, severe diabetic eye disease.

The variables of body composition most strongly associated with the presence of SDED were the visceral fat/android fat mass ratio (OR 1.40 per 0.1 increase, *z* = 3.13), followed by the android fat/appendicular lean mass ratio (OR 1.91 per 0.1 increase, *z* = 2.13) and the visceral fat/appendicular lean mass ratio (OR 1.16 per 0.01 increase, *z* = 2.45) (Table 4). The percentages of visceral and android fat were positively associated with SDED, whereas the percentage of appendicular lean mass was negatively associated with SDED (Table 4). Interestingly, the percentages of the total body fat and the total body lean masses were not associated with SDED (Table 4). Using the “no previous retinopathy” in the FinnDiane questionnaire as the reference group in a multinominal logistic regression model, the visceral fat mass percentage was also associated with any retinopathy except SDED at baseline (OR 1.63, 95% CI 1.03-2.59, *P *= 0.04); however, the total body fat mass percentage was not (OR 1.01, 95% CI 0.97-1.05, *P *= 0.62). The associations between body composition and SDED are shown in Table 4.

**Table 4.** Associations between body composition and the prevalence of severe diabetic eye disease

Body composition	OR (95%CI)	z-value	P-value
Visceral fat/android fat mass ratio, per 0.1 increase	1.40 (1.13-1.73)	3.1287	0.002
Android fat/appendicular lean mass ratio, per 0.1 increase	1.91 (1.05-3.47)	2.1328	0.03
Visceral fat/appendicular lean mass ratio, per 0.01 increase	1.16 (1.03-1.31)	2.4478	0.01
Visceral fat mass percentage, per 1% increase	1.80 (1.12-2.88)	2.4466	0.04
Android fat mass percentage, per 1% increase	1.28 (1.03-1.59)	2.0813	0.03
Appendicular lean mass percentage, per 1% increase	0.93 (0.84-1.02)	-1.5030	0.13
Total body lean mass percentage, per 1% increase	0.97 (0.93-1.01)	-1.3088	0.19
Total body fat mass percentage, per 1% increase	1.03 (0.99-1.07)	1.3010	0.19

The logistic regression model was adjusted for age at onset of diabetes, duration of diabetes, sex, glycated hemoglobin A1c, systolic blood pressure, triglycerides, smoking, lipid-lowering medication, estimated glomerular filtration rate, and diabetic nephropathy stage. Appendicular means both arms and legs. The percentage of body fat and lean mass are related to total body weight.

Abbreviation: OR, odds ratio.

## Discussion

In this study, we showed that a simple measure such as the WHtR is associated with an increased risk of SDED in adults with T1D, placing it among the 6 most important risk factors for SDED in this population. Furthermore, we found that the central body fat distribution is associated with the presence of SDED. We are not aware of any other studies in a large cohort of individuals with T1D that have assessed such relationships, especially stratified by different stages of albuminuria.

Obesity is causally related to DN in individuals with T1D (17), while its relationship with SDED is still unclear. Although studies including individuals with type 2 diabetes have shown that a higher BMI was associated with DR (18,19), a meta-analysis and systematic review revealed that being overweight or obese did not confer an increased risk of DR (20). Possibly, these discrepancies concerning the relationship between BMI and DR in individuals with type 2 diabetes are because BMI does not necessarily reflect the body fat distribution, especially the central fat, which has been associated with DR in people with type 2 diabetes (10,11). Concerning studies in individuals with T1D, the data are even more scarce, and the results are also controversial. Similar to our findings, a Belgian cross-sectional study (6), including 592 participants with T1D, and the DCCT/EDIC study (3) have shown that individuals with DR presented with a higher BMI. A cross-sectional Australian study, including 501 adults with T1D, found an association between the BMI > 30kg/m^2^ and DR (7). However, in the DCCT/EDIC study (3) the authors did not find an association between BMI and the progression of DR. Nevertheless, we have to take into consideration that we are looking at different endpoints. The DCCT/EDIC study evaluated the progression of DR, while the FinnDiane study did not look at each progressive stage of DR but rather at the risk of developing a severe stage of diabetic eye disease. The discrepancies also may be explained by the fact that the DCCT/EDIC cohort is better clinically characterized, including a greater number of individuals with ETDRS grading than the FinnDiane cohort. Another possible reason may be related to the relationship between body composition and SDED, which was not explored in the DCCT/EDIC study. Since we showed that the visceral fat mass percentage but not the total body fat mass percentage is associated with SDED, differences in the body composition between the FinnDiane cohort and DCCT/EDIC cohort may explain different results, despite a similar BMI. In our study, BMI was positively associated with SDED, although it was the third of 4 anthropometric measures in the ranking of relevance. The weaker association, by *z* value, between BMI and SDED compared to the association between WHtR and SDED may be due to the lower power of BMI compared to WHtR to estimate the visceral fat in individuals with T1D, according to previous research of our group (21). Recently, we also showed that although BMI and WHtR are associated with nonalcoholic fatty liver in adults with T1D, WHtR shows a stronger association than BMI (22). The observed differences between BMI and WHtR are even more relevant in clinical practice since, given that in the present data set, 10.7% of the individuals with normal BMI and 69.1% of those with higher than normal BMI presented a WHtR ≥ 0.5, which means that several individuals at high risk of SDED would not be recognized if only a BMI ≥ 30kg/m^2^ is considered as a risk factor.

Central fat, estimated by WHR, has been associated with DR in a few studies including individuals with type 2 diabetes (10,11). However, in the present study, it was the last of 4 anthropometric measures in the ranking of relevance, and beyond that, WHR was not associated with SDED in the subgroup analysis according to DN stages. To understand the disagreement with the literature, it is important to recognize that the present study included individuals with T1D, which differs from those with type 2 diabetes in many aspects. Furthermore, according to our previous research, WHR is inferior to WHtR as an estimator of visceral fat (21). In other words, it seems that visceral fat is the main factor for SDED; therefore, the stronger the association between the anthropometric measure and visceral fat is, the better predictor it is.

In the present study, we showed for the first time that the percentage of visceral fat mass is closely associated with SDED in individuals with T1D and that the ratio of visceral to android fat shows an even stronger association. This result emphasizes the greater relevance of the visceral fat for the risk of SDED compared to the android fat, which includes the visceral and subcutaneous fat located at the android region. Furthermore, the associations between SDED and the ratios of visceral and android fat to appendicular lean mass demonstrate the importance of having a balance in the body composition concerning lean mass to central fat mass, since the functional muscle tissue improves insulin sensitivity whereas visceral fat increases insulin resistance. The mechanism involved in the relationship between visceral fat and SDED is still unknown, albeit some hypotheses can be suggested. Adipocytes from visceral fat produce plasminogen activator inhibitor type 1 (23), which has been associated with end-stage proliferative DR in individuals with type 2 diabetes (24). Visceral fat also produces tumor necrosis factor alpha (25), which has been associated with DR in individuals with T1D (26), as well as leading to an inflammatory and insulin-resistant state (27), thus contributing to the increase in blood glucose and triglycerides, 2 relevant risk factors for SDED. Since insulin resistance has also been associated with low skeletal muscle mass (28), which has been associated with DR in type 2 diabetes (29), it may explain the negative association between SDED and the appendicular lean mass percentage as well as the positive association between SDED and the ratios of central fat to appendicular lean mass in our study. Another possible link between SDED and visceral fat is the positive association between visceral fat and VEGF (30), which is involved in the pathogenesis of DR (31).

Another novelty of the present study was to show the contribution of WHtR alongside the well-known risk factors for SDED. Similarly to the results from the DCCT/EDIC study (3), we showed that the HbA1c is the most important risk factor for SDED in our cohort. However, we also found that central obesity, represented by WHtR, is another important risk factor. It is of note that the association between WHtR and the risk of SDED remained after adjusting for ETDRS grading in a subset of individuals.

In this study, no anthropometric measure was associated with the risk of SDED in individuals with macroalbuminuria. Possibly, the advanced DN stage is such an important risk factor for SDED that it overwhelms any other risk factor.

The present study has some limitations. We used the variable “any retinopathy at baseline” to adjust the analysis, but there was no detailed information on the grading of the retinopathy in the questionnaires. Another limitation is that we did not have information on ETDRS grading for all individuals at baseline and during FinnDiane follow-up visits, which hampers any assessment of the impact of body composition and WHtR on the progression of DR, as was done with BMI in the landmark DCCT/EDIC study. However, we tried to mitigate this limitation by performing a sensitivity analysis that showed that WHtR was still associated with SDED after adjusting for baseline ETDRS grading. Another limitation of the present study is the fact it was conducted in a Caucasian-Finnish population with T1D; therefore, we cannot exclude whether ethnicity may have an impact on the results, since the waist threshold may differ according to ethnicity. On the other hand, the WHtR threshold of 0.5 we found in our cohort for the risk of SDED was the same well-known WHtR threshold for cardiovascular risk and mortality (15,32) in the general population. Thus, our findings may motivate further studies to investigate the mechanisms involved in the relationship between visceral fat and SDED. This study has several strengths and the main one is the long-term follow-up of a large cohort of individuals with T1D. Second, the body composition was assessed by DXA, which is the gold standard method. Furthermore, we showed in a large sample of individuals with T1D that WHtR, a simple measure with a unique threshold for both sexes, is associated with the risk of a severe complication of diabetes in the absence and at the early stage of DN. From a clinical perspective, this study not only highlights a new modifiable risk factor for SDED, but more important, it shows that a simple anthropometric measure related to central obesity is associated with SDED in individuals with T1D. Given that WHtR is a modifiable risk factor, our results reinforce the relevance of treating central obesity beyond blood glucose in individuals with T1D.

In conclusion, the central distribution of body fat is associated with SDED, and WHtR, a hallmark of central obesity, is associated with an increased risk of developing SDED in adults with T1D. This study supports the inclusion of WHtR beyond BMI in the routine consultation of individuals with T1D.

## Data Availability

Restrictions apply to the availability of some or all data generated or analyzed during this study to preserve patient confidentiality or because they were used under license. The corresponding author will on request detail the restrictions and any conditions under which access to some data may be provided.

## Additional Information

**Disclosures:** E.B.P. reports receiving lecture honorariums from Eli Lilly, Abbott, Astra Zeneca, Sanofi, and Boehringer Ingelheim and is an advisory board member of Sanofi. P-H.G. reports receiving lecture honorariums from Astellas, Astra Zeneca, Boehringer Ingelheim, Eli Lilly, Medscape, MSD, Mundipharma, Novo Nordisk, PeerVoice, and Sanofi, Sciarc and being an advisory board member of Astellas, Astra Zeneca, Bayer, Boehringer Ingelheim, Eli Lilly, Janssen, Medscape, MSD, Mundipharma, Novo Nordisk, and Sanofi. No other potential conflicts of interest relevant to this article were reported. V.H. and C.F. report no conflict of interest.

## References

[1] Flaxman SR , Bourne RRA, Resnikoff S, et al. Global causes of blindness and distance vision impairment 1990-2020: a systematic review and meta-analysis. Lancet Glob. Health. 2017;5(12):e1221–e1234. 29032195 10.1016/S2214-109X(17)30393-5

[2] Lammi N , Blomstedt PA, Moltchanova E, Eriksson JG, Tuomilehto J, Karvonen M. Marked temporal increase in the incidence of type 1 and type 2 diabetes among young adults in Finland. Diabetologia. 2008;51(5):897-899. 18317725 10.1007/s00125-008-0952-9

[3] Hainsworth DP , Bebu I, Aiello LP, et al. Risk factors for retinopathy in type 1 diabetes: the DCCT/EDIC study. Diabetes Care 2019;42(5):875–882. 30833368 10.2337/dc18-2308 PMC6489114

[4] Hietala K , Wadén J, Forsblom C, et al.; FinnDiane Study Group. HbA1c variability is associated with an increased risk of retinopathy requiring laser treatment in type 1 diabetes. Diabetologia. 2013;56(4):737-745. 23314044 10.1007/s00125-012-2816-6

[5] Hietala K , Harjutsalo V, Forsblom C, Summanen P, Groop PH; FinnDiane Study Group. Age at onset and the risk of proliferative retinopathy in type 1 diabetes. Diabetes Care. 2010;33(6):1315-1319. 20185730 10.2337/dc09-2278 PMC2875446

[6] Block CEMD , Leeuw IHD, Gaal LFV. Impact of overweight on chronic microvascular complications in type 1 diabetic patients. Diabetes Care. 2005;28(7):1649-1655. 15983315 10.2337/diacare.28.7.1649

[7] Price SA , Gorelik A, Fourlanos S, Colman PG, Wentworth JM. Obesity is associated with retinopathy and macrovascular disease in type 1 diabetes. Obes Res Clin Pract. 2014;8(2):e178-e182. 24743014 10.1016/j.orcp.2013.03.007

[8] Ross R , Neeland IJ, Yamashita S, et al. Waist circumference as a vital sign in clinical practice: a consensus statement from the IAS and ICCR working group on visceral obesity. Nat Rev Endocrinol. 2020;16(3):177-189. 32020062 10.1038/s41574-019-0310-7 PMC7027970

[9] Parente EB . Is body mass index still a good tool for obesity evaluation? Arch Endocrinol Metab. 2016;60(6):507-509. 27982197 10.1590/2359-3997000000232 PMC10522173

[10] Raman R , Rani PK, Gnanamoorthy P, Sudhir RR, Kumaramanikavel G, Sharma T. Association of obesity with diabetic retinopathy: Sankara nethralaya diabetic retinopathy epidemiology and molecular genetics study (SN-DREAMS Report no. 8). Acta Diabetol. 2010;47(3):209-215. 10.1007/s00592-009-0113-8 19326040

[11] Man RE , Sabanayagam C, Chiang PP, et al. Differential association of generalized and abdominal obesity with diabetic retinopathy in Asian patients with type 2 diabetes. JAMA Ophthalmol. 2016;134(3):251-257. 26720805 10.1001/jamaophthalmol.2015.5103

[12] Dahlström EH , Sandholm N, Forsblom CM, et al. Body mass index and mortality in individuals with type 1 diabetes. J Clin Endocrinol Metab. 2019;104(11):5195-5204. 31034018 10.1210/jc.2019-00042

[13] Davis MD , Fisher MR, Gangnon RE, et al. Risk factors for high-risk proliferative diabetic retinopathy and severe visual loss: early treatment diabetic retinopathy study report #18. Invest Ophthalmol Vis Sci. 1998;39(2):233-252. 9477980

[14] Meredith-Jones K , Haszard J, Stanger N, Taylor R. Precision of DXA-derived visceral fat measurements in a large sample of adults of varying body size. Obesity. 2018;26(3):505-512. 29286209 10.1002/oby.22108

[15] Browning LM , Hsieh SD, Ashwell M. A systematic review of waist-to-height ratio as a screening tool for the prediction of cardiovascular disease and diabetes: 0·5 could be a suitable global boundary value. Nutr Res Rev. 2010;23(2):247-269. 20819243 10.1017/S0954422410000144

[16] Meyers J , Mandrekar J. Cutpoint Determination methods in survival analysis using SAS®: updated %FINDCUT macro. In: SAS Conference Proceedings. SAS Institute Inc; 2015.

[17] Todd JN , Dahlström EH, Salem RM, et al; FinnDiane Study Group. Genetic evidence for a causal role of obesity in diabetic kidney disease. Diabetes. 2015;64(12):4238-4246. 26307587 10.2337/db15-0254 PMC4657582

[18] Dirani M , Xie J, Fenwick E, et al. Are obesity and anthropometry risk factors for diabetic retinopathy? The diabetes management project. Invest Ophthalmol Vis Sci. 2011;52(7):4416-4421. 21482643 10.1167/iovs.11-7208

[19] van Leiden HA , Dekker JM, Moll AC, et al. Blood pressure, lipids, and obesity are associated with retinopathy: the Hoorn study. Diabetes Care 2002;25(8):1320-1325. 12145228 10.2337/diacare.25.8.1320

[20] Zhou Y , Zhang Y, Shi K, Wang C. Body mass index and risk of diabetic retinopathy: a meta-analysis and systematic review. Medicine. 2017;96(22):e6754. 28562529 10.1097/MD.0000000000006754 PMC5459694

[21] Parente EB , Mutter S, Harjutsalo V, Ahola AJ, Forsblom C, Groop PH. Waist-height ratio and waist are the best estimators of visceral fat in type 1 diabetes. Sci Rep. 2020;10(1):18575. 33122731 10.1038/s41598-020-75667-5 PMC7596092

[22] Parente EB , Dahlström EH, Harjutsalo V, et al. The relationship between body fat distribution and nonalcoholic fatty liver in adults with type 1 diabetes [published online ahead of print, 2021 May 24]. Diabetes Care. Pubished online May 2021. doi:10.2337/dc20-3175 10.2337/dc20-3175 34031143

[23] Alessi MC , Peiretti F, Morange P, Henry M, Nalbone G, Juhan-Vague I. Production of plasminogen activator inhibitor 1 by human adipose tissue: possible link between visceral fat accumulation and vascular disease. Diabetes. 1997;46(5):860-867. 9133556 10.2337/diab.46.5.860

[24] Zhong ZL , Chen S. Plasma plasminogen activator inhibitor-1 is associated with end-stage proliferative diabetic retinopathy in the Northern Chinese Han population. Exp Diabetes Res. 2012;2012:350852. 23304115 10.1155/2012/350852 PMC3518968

[25] Halberg N , Wernstedt-Asterholm I, Scherer PE. The adipocyte as an endocrine cell. Endocrinol Metab Clin North Am. 2008;37(3):753-68, x. 18775362 10.1016/j.ecl.2008.07.002 PMC2659415

[26] Yao Y , Li R, Du J, et al. Tumor necrosis factor-α and diabetic retinopathy: review and meta-analysis. Clin Chim Acta. 2018;485:210-217. 29959897 10.1016/j.cca.2018.06.028

[27] Bertin E , Nguyen P, Guenounou M, Durlach V, Potron G, Leutenegger M. Plasma levels of tumor necrosis factor-alpha (TNF-alpha) are essentially dependent on visceral fat amount in type 2 diabetic patients. Diabetes Metab. 2000;26(3):178-182. 10880890

[28] Cleasby ME , Jamieson PM, Atherton PJ. Insulin resistance and sarcopenia: mechanistic links between common co-morbidities. J Endocrinol. 2016;229(2):R67-R81. 26931135 10.1530/JOE-15-0533

[29] Fukuda T , Bouchi R, Takeuchi T, et al. Association of diabetic retinopathy with both sarcopenia and muscle quality in patients with type 2 diabetes: a cross-sectional study. BMJ Open Diabetes Res Care. 2017;5(1):e000404. 10.1136/bmjdrc-2017-000404 PMC5530250 28761661

[30] Miyazawa-Hoshimoto S , Takahashi K, Bujo H, Hashimoto N, Saito Y. Elevated serum vascular endothelial growth factor is associated with visceral fat accumulation in human obese subjects. Diabetologia. 2003;46(11):1483-1488. 14534780 10.1007/s00125-003-1221-6

[31] Jampol LM , Glassman AR, Sun J. Evaluation and care of patients with diabetic retinopathy. N Engl J Med. 2020;382(17):1629-1637. 32320570 10.1056/NEJMra1909637

[32] Jayedi A , Soltani S, Zargar MS, Khan TA, Shab-Bidar S. Central fatness and risk of all cause mortality: systematic review and dose-response meta-analysis of 72 prospective cohort studies. BMJ. 2020;370:m3324. 32967840 10.1136/bmj.m3324 PMC7509947

